# Supplementary material for: Can’t simply roll it out: Evaluating a real-world virtual reality intervention to reduce driving under the influence
Source: PLoS One. 2021 Apr 29;16(4):e0250273. doi: 10.1371/journal.pone.0250273 (PMC8084189; doi:10.1371/journal.pone.0250273)
Supplement: S2 Appendix — (PDF) [file pone.0250273.s002.pdf]

Open Youth Institute for Research, Education and Development

Институт за изследвания, образование и развитие Отворена младеж

Evaluation Plan

Daniel Vankov

d.vankov@ired-bg.eu

Date 28/02/2019

Status: Final

Virtual reality and real volunteers for Advancing Safer Traffic participation for young people

Project No: 587544-EPP-1-2017-1-BE-EPPKA2-CBY-ACPALA

Acronym of the Project: VAST

Co-funded by the
Erasmus+ Programme
of the European Union

This project has been funded with support from the European Commission. This evaluation plan reflects the views only of the author, and the Commission cannot be held responsible for any use which may be made of the information contained therein.

Contents

List of tables and figures 5

1. Project rationale 6

2. Project partners 7

3. Aims and objectives 7

4. Summary of findings 9

5. Evaluation methodology overview 11

5.1 General description 11

5.2 Project activities 12

5.3 Tasks and measurable indicators 14

5.4 Overview of VAST evaluation 15

6. Evaluation 1: Preparation activities 17

6.1 QUE 1: Questionnaire at the Kick-off meeting 17

6.2 QUE 2: Questionnaire at the end of the Media capacities workshop series 18

6.3 QUE 3.1: Questionnaire at the first Project advisory groups meetings 21

6.4 QUE 4: Questionnaire at the National Youth Summits on Road Safety 23

7. Evaluation 2: Campaign implementation 24

7.1. QUE 5: Questionnaire regarding the national implementation plans of the Joint Campaign 26

7.2. QUE 6: Questionnaire regarding the national implementation of the Joint Campaign 29

7.3. QUE 7: Questionnaire regarding the national implementation of the Joint Campaign 33

7.3.1. Argentina 33

7.3.2. Belgium 33

7.3.3. Bulgaria 34

7.3.4. Netherlands 34

7.3.5. Romania 34

7.4. QUE 8: Questionnaire regarding the national implementation of the Joint Campaign 35

7.4.1. Theory of the Planned Behaviour 35

7.4.2. Evaluation design 36

7.4.3. Recruitment of participants 37

8. Evaluation 3: Dissemination activities 37

8.1. QUE 3.2: Questionnaire at the second Project advisory groups meetings 39

8.2. QUE 9: Questionnaire at youth workers' mobility 40
8.1. QUE 10: Questionnaire at the International conferences 42
8.1.1. Brussels, Belgium 42
8.1.2. Buenos Aires, Argentina 43
9. Conclusions and suggestions 44

List of tables and figures

Table 1.1. Age groups: share of all road deaths and share of total population (Source: EC) 6
Table 5.1. VAST process methodology timeline with milestones (Q – quarter) 14
Table 5.2. Time-line of the evaluation 16
Table 6.1. Overview of the questionnaires in evaluation phase 1 17
Table 6.2. Feedback from youth exchange participants in regards to the project design............................... 18
Table 6.3. Clarity of the future work post workshops 21
Table 6.4. Feedback from PAG members in regards to the project design.. 22
Table 6.5. Feedback from National Summits on Road Safety (scales from 1, worse, to 5, best) 23
Table 7.1. Overview of the questionnaires in evaluation phase 2 24
Table 7.2. Implementation plans of the Joint International Road Safety Campaign...................................... 26
Table 7.3. Campaign implementation Argentina, Belgium and Bulgaria 29
Table 7.4. Campaign implementation in the Netherlands and Romania 30
Table 7.5. Dissemination activities during the campaign implementation 32
Table 7.6. Measures and time of measurement. 36
Table 7.7. Number of collected participants' questionnaires per country, time and condition. 37
Table 8.1. Overview of the questionnaires in evaluation phase 3 37
Table 8.2. Feedback from PAG members in regards to the project design.. 39
Table 8.3. Feedback from the youth workers' mobilities participants... 40
Table 8.4. European Road Safety Summit feedback 43
Table 8.5. South American Road Safety Summit feedback 44

Figure 1.1. Fatalities since 2000 (Source: EC).. 6
Figure 5.1. Overview of the 3-evaluations approach 15
Figure 6.1. Workshops participants per country and role in VAST.. 19
Figure 6.2. Why do you want to participate in the VAST?... 19
Figure 6.3. Main means of transport of the workshops' participants. 20
Figure 6.4. Who do you discuss risky behaviour in public traffic with? 20
Figure 6.5. First meeting PAG participants per country. 22
Figure 7.1. Theory of planned behaviour 35
Figure 8.1. Second meeting PAG participants per country. 39

1. Project rationale

"The EU target *[of halving the road deaths by 2020]* is at stake. ... At the EU and national level, there has been a distinct lack of action. ... 2015 was the second consecutive poor year for road safety; 26,300 people lost their lives on EU roads in 2015 compared to 25,970 in 2014, representing an increase of 1%" (Figure 1.1) (ETSC, June 2016).

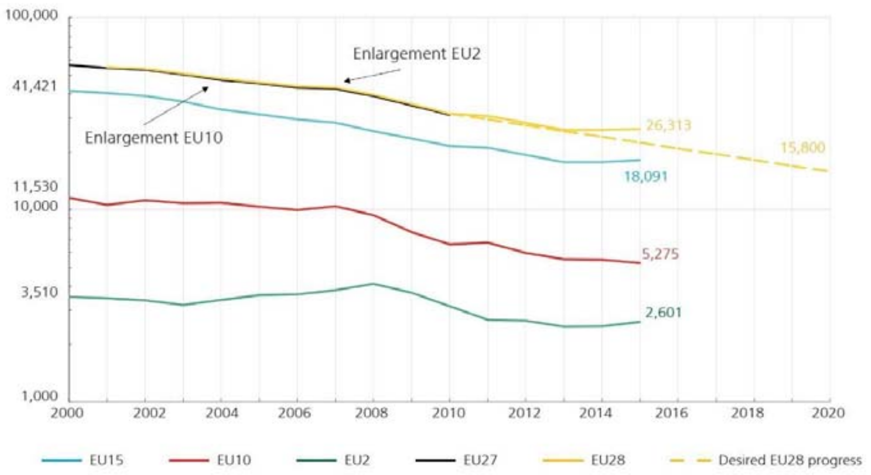

Figure 1.1. Fatalities since 2000 (Source: EC)

"Road traffic injuries are the eighth leading cause of death globally, and the leading cause of death for young people aged 15–29." (WHO, 2015). The European Commission (EC) states "The young people, between 15 and 24 years old, make up 11% of the population but 17% of all road fatalities. This means that the young still face the largest risk in traffic." (See Table 1.1) (Road Safety in European Union (RSEU), March 2015)

Table 1.1. Age groups: share of all road deaths and share of total population (Source: EC)

AGE GROUP	% OF FATALITIES	% OF POPULATION
<15	<3	16
15-24	17	11
25-49	36	35
50-64	19	20
>65	25	18

"In the Region of the Americas, road traffic injuries are the leading cause of death in children aged 5–14 and the second leading cause in the group aged 15–44." (Road safety facts in the regions of the Americas, WHO, 2013).

Drink-driving is identified as a main contributor to 30% of the fatal and 9% of the non-fatal injuries (ATC, 2011). Still research shows that 7.8% of the young driver respondents had driven after drinking alcohol while 20% rode with a driver who had been drinking (CDCP, 2016). Drug-driving is a main behavioral factor in 7% of the fatal and 2% of the non-fatal crashes (ATC, 2011).

These findings lead the project partners to two conclusion:

- Current actions play their role and need to stay in place; and

- New actions need to be developed to support reaching the EU targets.

Such unacceptably high level of fatalities and injuries caused by completely preventable voluntary risky behaviour *(young people's alcohol and drug use when driving)* negatively influences many young people's daily lives in relation to health, education, employment and well-being. This is why one of the VAST objectives was achieving a real and measurable behavioural change in the young people's (aged 16-25, incl. ones with less opportunities) attitudes towards drink/drug-driving. The VAST project promoted cooperation between stakeholders with the goal to help young people (incl. ones with fewer opportunities) grow and play an active part in their society by making safer driving an integral part of their life. This is aligned with the objectives of the Europe 2020 strategy and its flagship initiatives. They aim at supporting the effective use of human and social capital by improving its health and providing opportunities for both youth workers and young people to acquire new skills, knowledge and experience through informal education, mobility and cooperation. The project supported the EU Youth Strategy in promoting dialogue between youth and policy makers. VAST increased active citizenship, fostered social integration, and ensured their inclusion in policy development. Through the planned activities, VAST explored synergies between the fields of informal education, training and youth. It provided a platform for exceptional civil society players from Argentina, Belgium, Bulgaria, the Netherlands and Romania to continue their outstanding activities and leading role in helping their countries reach targets in the fields of health, education and employment.

2. Project partners

VAST involved five project partners, four from the European Union and one from South America

Leading partner: Responsible Young Drivers Vlaanderen vzw (RYD BE), Belgium

Partners:

- Narodno Chitalishte "Svetal Den 2009" (NCSD), Bulgaria
- Stichting Responsible Young Drivers Nederland (RYD NL), the Netherlands
- Asociatia Pentru Tinerii si Studentii din Partium (PIHE), Romania
- MiNU Asociacion Civil (MiNU), Argentina

3. Aims and objectives

VAST aimed to embrace the potential of next generation virtual reality technologies in an effort to address the overrepresentation of young people in road crashes.

An unfolded potential to build on the project partners' massive experience in deploying Information technologies through peer-to-peer communication strategies was identified at the beginning of VAST.

VAST used the advancement and availability of virtual reality experience as a leverage. A tailored International campaign specifically designed to engage and appeal to contemporary and future generations of drivers was designed to harvest the power of those technologies for social and public good. In the broader perspective it is hoped that the project contributed to the participating countries reaching their targets of reducing road fatalities in relation to the European Road Safety Policy Orientations 2011-2020 and the UN "Decade of Action on Road Safety (2011- 2020)".

The VAST objectives were to:

1. Achieve a real and measurable behavioral change in the young people's attitudes towards drink/drug-driving (incl. ones with less opportunities). The Joint International Campaign on Road Safety was geared to reach out to young people with social, economic and geographical difficulties. A robust psychological methodology was used to evaluate if actual behavioral change was achieved due to the campaign implementation.

2. Develop a higher level of cooperation between the partners through capacity building by:

- Going from cross-fertilization of experience to a joint peer-to-peer campaign. A Joint International Campaign on Road Safety was implemented and evaluated in each of the partner countries.
- Involving a substantial number of youth workers (two per partner) in the partners' meetings, the youth workers' mobilities and the European and South American Youth Road Safety Summits.
- Establishing grounds for significant conventional and social media presence. Youth workers and young volunteers (aged 18 to 25) were extensively trained in media relations through three different thematic workshops in each country. PR experts supported campaign implementations.

3. Directly involve a massive number of young people:

- 25 young volunteers in trainings and implementing campaigns;
- 250 young people in the National Youth Summits on Road Safety;
- 3000 direct participants (600 per country) in the Joint International Campaign on Road Safety;
- 35 young people/youth workers participants in the European (24) and South American (11) Road Safety Summits.

4. Provide basis for increased involvement of road safety stakeholders and high-level lobbying for better road safety policy development through:

- Establishment of five Project Advisory Groups (PAG). Each PAG was expected to consist of 5 to 10 people, representing state or regional government institutions, academia, business and other road safety NGOs. At least two PAG meetings were to be held in each country.

- Involving recognised International speakers in the European and South American Youth Road Safety Summits. Each hosting partner had travel and subsistence budget to support the participation of two International experts (1 from the region and 1 from overseas) outside the project consortium.

4. Summary of findings

The VAST project succeeded in fulfilling its aims and objectives as described below. All country coordinators reported successful implementation of the Joint International Road Safety Campaign. The project foresaw 3,000 participants, 600 per country, to be directly involved. The target was overpassed with more than 50%, based on the reported 5,301 directly reached people. Additional 202,797 people were reached indirectly. Both numbers reveal that the partners' expected outreach was overpassed, too. Initially, they planned to reach a total of 2,950 people.

The campaigns were implemented by 20 youth workers and 31 young volunteers. The initial plan was to involve 52 youth workers and volunteers so the estimation was very close to the actual number. Thus the partners were able to get enough on the ground which ultimately translated into the better than initially planned outreach. It is an interesting observation that this success is not due to a much higher number of actions than initially expected. The partners planned to implement 89. However, the final number was 93. It has to be noted that 30 working days were envisaged for tailored actions such as bringing the virtual reality simulator to local events, answering invitations from interested parties, and participation in school/university road safety days or implementing open days at the partners' own premises. However, there was no minimum or a maximum number of working days per action. Only one problem was reported during implementation and it was resolved in a due term.

Overall, the country coordinators reported a rating of 6.20/7.00 on their satisfaction with the implementation and a little lower 6.00/7.00 satisfaction, perceived in the directly reached participants.

Unfortunately, not all youth workers and volunteers, reported by the country coordinators provided their feedback. In total 45 did (18 youth workers and 27 volunteers, 22 females and 23 males, average age 24 years). They reported being directly involved in a total of 295 actions. The number is higher than the one reported by the country coordinators and the likely reason for that is an overlap, e.g. youth workers and volunteers engaging with the same participants.

It is interesting to see that youth workers and volunteers were less critical than the country coordinators and reported a rating of 6.60/7.00 on their satisfaction with the implementation and a little lower 6.47/7.00 satisfaction, perceived in the directly reached participants. Both ratings are overly positive and hint for a job well done by not only the country coordinators but by the youth workers and volunteers themselves. 75% thought the campaign will help make the young people safer traffic participants. 82% thought the campaign could not have been carried out with similar success but without the Virtual reality equipment. The overall rating of the impact of the virtual reality simulation on the involved participants was 6.49/7.00.

These very high ratings and confirmation of impact from the directly involved people were confirmed at large by the project external audience during the two implemented International Summits. The VAST objectives were rated as clear with 4.55/5.00 in Buenos Aires and 4.30/5.00 in Brussels. They were also rated as achievable with 4.38/5.00 in Buenos Aires and 4.27/5.00 Brussels. The project working steps were rated as clear with 4.45/5.00 in Buenos Aires, and 4.30/5.00 in Brussels. Conference participants expected it would produce a lasting change in the participating young people with 4.49/5.00 in Buenos Aires and 3.76/5.00 in Brussels. VAST received an overall rating in respect of its design, implementation, impact, sustainability of 4.18/5.00 in Buenos Aires and 3.72/5.00 in Brussels. 92% of the Summits' participants said it was successfully implemented. The other 8% thought the success was to a certain extent.

The project expected results, mentioned in the project proposals, were:

1. Achieve a real and measurable behavioural change in the young people's attitudes towards drink/drug-driving (incl. ones with less opportunities).

It was not possible to test for such an effect due to the lack of quality data, required by the intended statistical tests.

2. Develop and implement a Joint International Campaign on Road Safety.

Accomplished! The campaign was implemented by the project partners in Argentina, Belgium, Bulgaria, the Netherlands and Romania.

3. Involving a substantial number of youth workers (two per partner) in the partners' meetings, the youth workers' mobilities and the European and South American Youth Road Safety Summits.

Accomplished! Eight youth workers provided feedback as being involved in the youth workers' mobilities in Belgium, 7 – in Argentina.

4. Train 40 youth workers and young volunteers (aged 18 to 25) in media relations through three different thematic workshops in each country.

Accomplished! 49 people took part in the workshops (43 in "Media relations", 41 in "Social media management" and 44 in "Public relations and event management").

5. Directly involve 25 young volunteers in trainings and implementing campaigns.

Accomplished! 31 volunteer were trained in the media workshops. 27 took part in implementing campaigns.

6. Directly involve 250 young people in the National Youth Summits on Road Safety.

Accomplished! 273 people provided feedback after the National Youth Summits on Road Safety.

7. Directly involve 3000 direct participants (600 per country) in the Joint International Campaign on Road Safety.

Accomplished! 5,301 people were reached directly through the campaigns.

8. Directly involve 35 young people/youth workers participants in the European (24) and South American (11) Road Safety Summits.

Accomplished! 37 of the delegates provided feedback after the European and 53 after the South American Road Safety Summit.

9. Involve between 25 and 50 road safety stakeholders in the Project Advisory Groups (PAG).

Accomplished! 48 stakeholders took part in the first PAG meeting, 38 – in the second one.

10. Involve recognised International speakers in the European and South American Road Safety Summits.

Accomplished!

5. Evaluation methodology overview

5.1 General description

iRED was subcontracted as an independent external project evaluator for the implementation of an integrated evaluation system that:

- analysed the level of implementation of the working programme;
- verified the achievement level of activities that could affect the implementation of each subsequent activity;
- collected information about the effectiveness of the work in each partner's context.

The current final evaluation report was developed based on the findings. A special focus of the evaluation is the long term effect of the campaign on the direct young participants in regards to assessing any shifts in their attitudes towards the risky behaviour of drink/drug driving. Psychological models were used to measure the success of the International Road Safety Campaign in regards to motivating safer behaviour in the campaign participants. The Theory of Planned Behaviour (TPB) (Ajzen, 1985, 1987, 1991) was selected as theoretical underpinning. Measures were taken before and three months post participation. The participants were incentivised to participate in the data collection before their participation by connecting the use of the virtual simulator with completing a survey. The follow-up survey did not offer incentives. A social media campaign was planned to be implemented at the beginning of the project in order to collect data from the general public through a survey, effectively establishing a baseline of a control group. The survey respondents were also contacted at the end of the campaign for second data collection. This was used to account for general shifts in people's attitudes due factors external to VAST.

5.2 Project activities

VAST facilitated adoption of innovation in every day prevention activities. It built on the partner consortium previous work, already done in putting together the project application, by adapting a proper implementation methodology for achieving the project objectives (See Table 5.1). The consortium implemented the following activities:

1. Establishing the project context for the innovation (Activity Leader (AL): RYD NL). The project partners sent two representative each to elaborate on the needs analysis, already done in preparing the project proposal, going into deeper details. In order to properly build the scope of the VAST Joint International Road Safety Campaign, the partners reviewed the "3D Tripping" Dutch experience during the project kick-off meeting, which was carried out in Amsterdam, the Netherlands.

2. Media capacities development (AL: NCSD). Each partner selected a group of high-potential volunteer multiplies to undergo a set of capacity-building workshops at youth workers' level together with the partners youth workers on the topics of "Media relations", "Social media management" and "Public relations and event management".

3. Virtual simulator assembly (AL: RYD NL). Instead of buying an off-the-shelf simulator and software, which is very expensive, the project partners bought the needed parts (powerful laptop, driving seat console, G29 steering wheel, Oculus rift goggles and TV) and assembled the machine. RYD NL offered the simulation software for free to the partnership.

4. Establishment of Project advisory groups (AL: NCSD). Each partner established a Project advisory group (PAG) of road safety stakeholders, such as representatives of state or regional government institutions, academia, business and other road safety NGOs. The VAST project was presented for discussion and suggestions for implementation on local level to the PAGs' first meetings. The subsequent National Youth Summits on Road Safety and Joint International Road Safety Campaign were introduced to the audience.

5. National Youth Summits on Road Safety (AL: PIHE). In an effort to prepare the public for the implementation of the Joint International Road Safety Campaign national large scale youth events were carried out in cooperation with the PAG members in the form of National Youth Summits on Road Safety. They were organized by the project young road safety volunteers and were open for active young people and youth workers who are interested in road safety. During the Summits, the project was presented to the general public and the media as well as relevant road safety issues weree discussed. The subsequent Joint International Road Safety Campaign was introduced together with the opportunities it was providing.

6. Implementation of the Joint International Road Safety Campaign (AL: RYD BE). A joint road safety intervention was carried out by the five partners through a series of activities effectively representing local actions with the new virtual reality simulators. The focus was on drink/drug-driving prevention for young

people through a powerful education method using virtual reality. In one-to-one peer sessions the campaign offered to the young people to experience "what it's like to drive a car whilst drunk, on Ecstasy, high on Cannabis and tripping on Magic Mushrooms" in a safe environment. Thus non-formal learning through self-experience, self-reflexion and peer discussions was encouraged. The campaigns were youth-led by the volunteers, trained in the media workshops, and supported by youth workers from the partner organizations. Each partner developed its own version of the campaign that best met local needs as defined by the PAGs and the National Youth Summits on Road Safety. Information material and give-aways were tailored to the local actions.

7. Second Project advisory groups' meetings (AL: PIHE). A second set of PAG meeting were carried out to share the campaign achievements and collect constructive feedback. During the meetings the results of the Joint International Road Safety Campaigns were presented. The subsequent European and South American Youth Summits on Road Safety were introduced.

8. European Road Safety Summit (AL: RYD BE). A European Road Safety Youth Summit was implemented to disseminate the project experience from the PAGs meetings, the National Youth Summits on Road Safety and the Joint International Road Safety Campaign in Europe. To enlarge the added value for the project partners, each was represented by two youth workers and their participation took the form of two-week youth workers mobility in support of the European Summit implementation for RYD BE. Young people and youth workers with interest in road safety youth work from European countries were invited to participate. The summit also featured road safety best practices from South America.

9. South American Road Safety Summit (AL: MiNU). A South American Road Safety Youth Summit was implemented to disseminate the project experience from the PAGs meetings, the National Youth Summits on Road Safety and the Joint International Road Safety Campaign in South America. To enlarge the added value for the project partners, each was represented by two youth workers and their participation took the form of two-week youth workers mobility in support of the South American Summit implementation for MiNU. Young people and youth workers with interest in road safety youth work from South American countries were invited to participate. The summit also featured road safety best practices from the European Union.

10. Final project meeting (AL: MiNU). A Final project meeting was held in Buenos Aires after the South American Road Safety Youth Summit during the youth workers' mobilities. During it besides purely technical tasks, such as preparation of final reports, the partners worked on preparing the project results and findings for international transferability by providing grounds for open access to its achievements and the results from its evaluation.

11. Project evaluation (AL: External). iRED was subcontracted as an independent project evaluator.

Table 5.1. VAST process methodology timeline with milestones (Q – quarter)

	2017		2018			
	Q3	Q4	Q1	Q2	Q3	Q4
Establishing the project context for the innovation (Kick-off meeting)	M1					
Media capacities development		M2				
Virtual simulator assembly		M3				
Establishment of Project advisory groups		M4				
National Youth Summits on Road Safety		M5				
Implementation of the Joint International Road Safety Campaign					M6, M7	
Second Project advisory groups' meetings					M8	
Youth workers' mobilities						M9
European Road Safety Youth Summit						M10
Youth workers' mobilities						M11
South American Youth Summit for Road Safety						M12
Final project meeting						M13
Project evaluation						M14

5.3 *Tasks and measurable indicators*

The following tasks were initially set for the project:

- Assemble 5 3D driving simulators;
- Implement a Kick-off meeting with 10 participants;
- Organize 5 local implementations of the Joint International Road Safety Campaign involving 3000 direct participants;
- Train 40 people on the topics of "Media relations", "Social media management" and "Public relations and event management" (15 workshops organized (3 per partner), 25 volunteer multipliers (5 per partner) and 15 youth workers (3 per partner) trained);
- Organize 16 youth workers' mobilities;
- Organise 10 PAG meetings;
- Involve 250 participants in 5 National Youth Summits on Road Safety;
- Involve 35 participants in the European Youth Road Safety Summit;
- Involve 25 participants in the South American Youth Road Safety Summit;
- Reach 10 000 people through information materials and 500 000 through conventional and social media.

5.4 *Overview of VAST evaluation*

The evaluation of VAST adopted a *formative* evaluation approach for the preparation and dissemination activities of the project and a *summative* approach for the implementation activities as demonstrated in Figure 5.1.

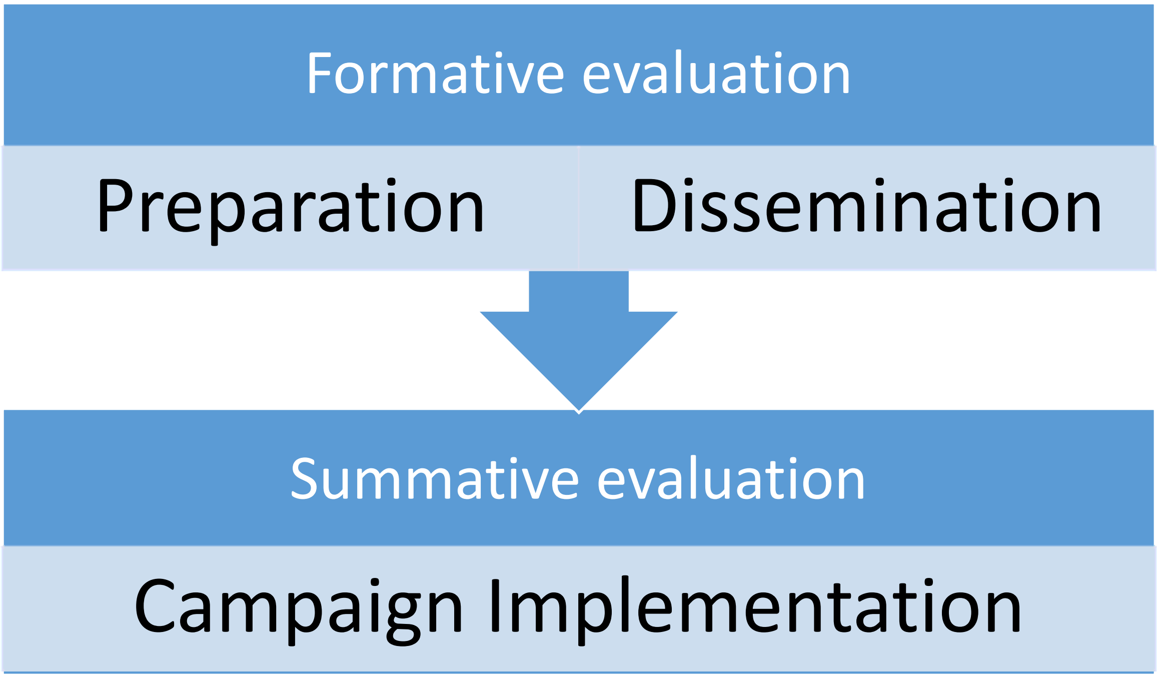

Figure 5.1. Overview of the 3-evaluations approach

The formative evaluation set outs a number of objectives to be achieved. Initially information was about the process of developing the Joint International Road Safety Campaign. Subsequently details were collected from each partner country regarding the implementation of the campaign on national level which was followed by an analysis to determine the long-term effect of the campaign on the involved participants. Following this, the evaluation aimed to verify the variables that affected the implementation and these shall serve to assist future endeavours. Finally, the evaluation set out to collect information about the effectiveness of the intervention in the different countries involved. It should be noted that the geographical location of the partner countries (Europe and South America) provided a wide coverage and hence allowed for a useful comparison to be made between different jurisdictions.

The main tool of the formative evaluation was implementation activities questionnaires which underlined the starting point and achievements of the project in relation to the target groups. The collected data was used as an information source for improving the project campaigns, communication tools and dissemination strategy. The evaluation methodology used a number of factors which serve as indicators of assessment/achievement throughout the project. These are as follows: the overall number of involved stakeholders through the PAGs, the overall number of young people involved both in the national summits and in the campaigns, the number of implemented activities (working days), the volume of traditional media

coverage, and the overall number of both young people and stakeholders involved in the International Youth Road Safety Summits.

This final evaluation includes assessing the project impact in each partner country. The evaluation shows the overall project achievements and can be used for dissemination activities on an International level.

The evaluation of VAST is conducted throughout the duration of the project (See Table 5.2) which is scheduled to run from 1st of July 2017 to 31st of December 2018. It was carried out consecutively as follows:

• **Evaluation 1** (EVA1 – formative evaluation): evaluation of the Preparation activities; started at the beginning of the project (July 2017, month 1) and lasted until the end of the National Youth Summits on Road Safety (December 2018, month 6).

• **Evaluation 2** (EVA2 – summative evaluation): evaluation of the Joint Campaign Implementation; started in January 2018 (month 7) and ended in August 2018 (month 14).

• **Evaluation 3** (EVA3 – formative evaluation): evaluation of the Dissemination activities; started in September 2018 (month 15) and ended in December 2018 (month 18).

Table 5.2. Time-line of the evaluation

	July 2017																	December 2018
	1	2	3	4	5	6	7	8	9	10	11	12	13	14	15	16	17	18
EVA1	■	■	■	■	■	■												
EVA2							■	■	■	■	■	■	■	■				
EVA3															■	■	■	■

6. Evaluation 1: Preparation activities

Table 6.1 gives an overview of the questionnaires which were used during the first evaluation phase.

Table 6.1. Overview of the questionnaires in evaluation phase 1

Action	Target group	Timing	Sample size	Questions regarding:
**EVALUATION 1: Preparation Activities – Formative approach**				
QUE 1: Questionnaire at the Kick-off meeting	Kick-off meeting participants	Month 4 – October 2017	10 in total	The general objectives of the project<br>Clarity and achievability of objectives<br>Clarity of working steps<br>Nature of the 3D simulator
QUE 2: Questionnaire at the end of the Media capacities workshop series	Youth workers and volunteer multiplies	Month 5 – November 2017	40 in total	Satisfaction with the organisation and content of the “Media relations”, “Social media management” and “Public relations and event management” workshops
QUE 3.1: Questionnaire at the first Project advisory groups meetings	Stakeholders / PAG participants	Month 5 – November 2017	25 in total	Up-to-date project activities<br>Added value to the project<br>Future activities
QUE 4: Questionnaire at the National Youth Summits on Road Safety	Participants	Month 6 – December 2017	250 in total	Up-to-date project activities<br>Added value to the project<br>Future activities

6.1 QUE 1: Questionnaire at the Kick-off meeting

The Kick-off meeting took place in Amsterdam, the Netherlands from 04 to 07.10.2017. The participating youth workers filled in a questionnaire to provide insights on whether the main objectives, working steps and activities, timing, etc. are fully clear. A total of 11 people (10 foreseen in the project application) returned completed questionnaires (3 from Argentina, 2 from Belgium, 2 from Bulgaria, 2 from the Netherlands and 2 from Romania). 10 were males and 1 was female. The average age was 34 years.

Table 6.2 the average score of the questions asked in regards to the clarity of the project is presented:

Table 6.2. Feedback from youth exchange participants in regards to the project design

Question	Average score (Likert scale 1=worse to 5=best)
Are the objectives of the project clear?	5.00
In your opinion, are the objectives achievable?	4.73
Are the working steps of the project clear?	4.82
Is the project timeframe appropriate?	4.18
Is your role in the project clear?	4.73

Average score values were skewed to the positive side of the scale. The issues regarding the objectives of the project, the participants` role in the project, envisaged working steps and the timeframe were completely clear.

The last questions explored whether the participants received enough information and experience in regards to the 3D virtual simulator. All participants said definite "Yes" providing the last piece of information that the Kick-off meeting had fulfilled its purpose.

6.2 QUE 2: Questionnaire at the end of the Media capacities workshop series

During the preparation phase of the VAST project a series of capacity-building workshops on the topics of “Media relations”, “Social media management” and “Public relations and event management” was organised by each partner in their respective country. During the sessions the participating youth workers and volunteers developed their own traffic safety campaign.

The participants of the workshops (youth workers and volunteers) were asked to fill in a questionnaire after the series was completed. A total of 49 people took part in the workshops (43 in "Media relations", 41 in "Social media management" and 44 in "Public relations and event management"). 40 were foreseen in the project application. See Figure 6.1 for a breakdown per country and role in the project.

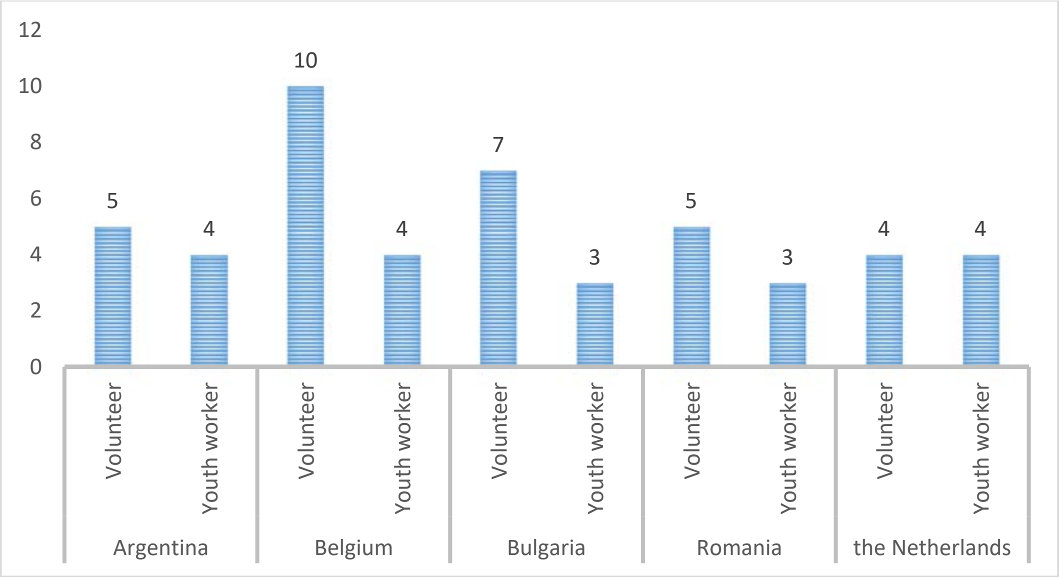

Figure 6.1. Workshops participants per country and role in VAST.

The questionnaire included questions about the youth workers and animators themselves (gender, age, background, motivation etc.), about the workshops (satisfaction with the training, critics etc.) and about the planning of the campaign (if the workshops helped, if they feel encouraged etc.).

The average age of the participants was 25 years. 30 of them were male, 18 were female and 1 preferred not to say. 27 reported to be currently occupied as employees, 12 as students, 8 as school pupils, 1 as a volunteer and 1 as an NGO Board member. They heard about the project and the workshops through their NGO (42 people), friends/acquaintances/fellow students (21 people), university/school (6 people) and by e-mail (5 people). The numbers add to more than 49 possibly because people received information from multiple sources. Figure 6.2 reveals their motivation to join the project.

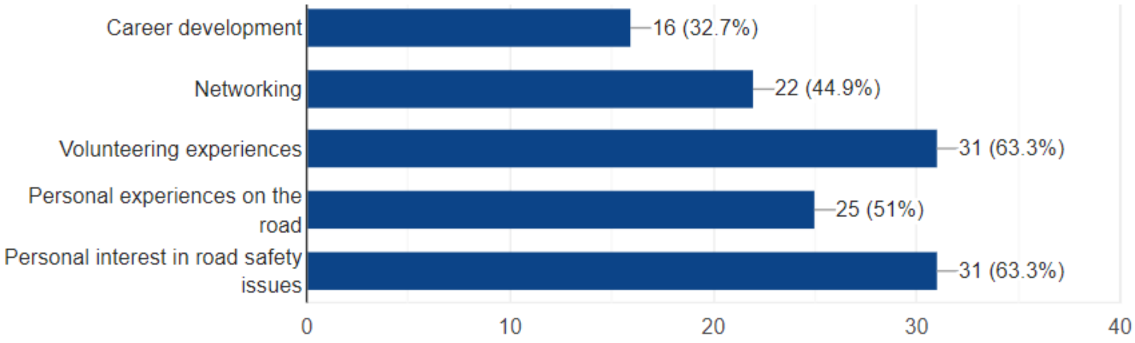

Figure 6.2. Why do you want to participate in the VAST?

The second part of the questionnaire helped us learn about the workshops' participants in relation to the broad theme of VAST, i.e. road safety. Figure 6.3 casts light on their modes of transport.

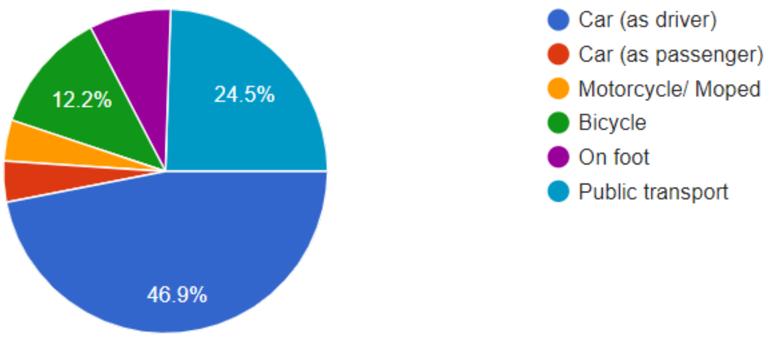

Figure 6.3. Main means of transport of the workshops' participants.

The collected data revealed that most of the participants were new to volunteering in a peer-to-peer campaigns. 37% reported as having experience in that. Even less, 27%, were experienced specifically in road safety campaigns. Nevertheless, the participants 1) consider themselves as being safe road users with an average rating of 4.18/5.00 and 2) they reported road safety to be a common topic of conversation. Figure 6.4 shows who the participants discuss risky behaviour in public traffic with.

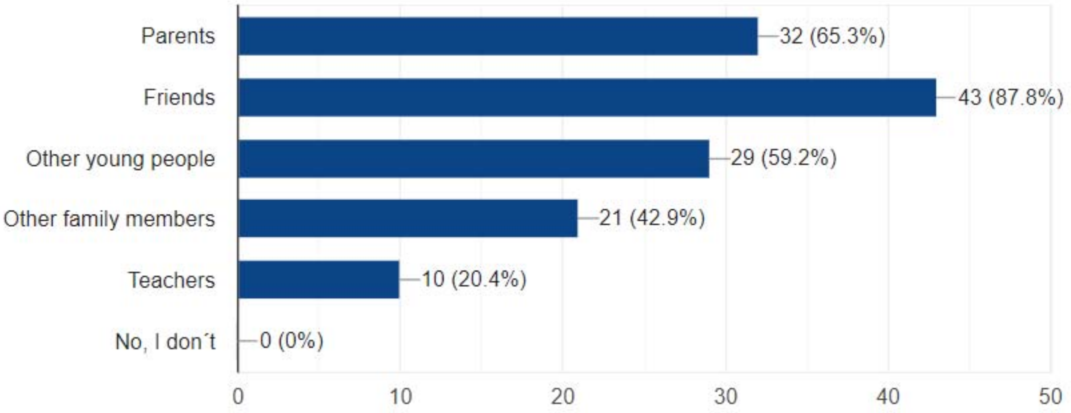

Figure 6.4. Who do you discuss risky behaviour in public traffic with?

The final set of questions explored the added value of the workshops. 98% reported that they have learned something new during the workshops. The overall satisfaction with the experience was rated at 4.27/5.00. Expectations were met with a rating of 4.25/5.00. When an opportunity to reflect on which part of the workshops they found most beneficial and why, the participants provided a multitude of answers, revealing the timeliness and added value of the activity. Here are some examples:

- *How to behave in front of camera and microphone.*
- *Everything was useful, because i did not work with media before.*
- *If I have to choose, I would say I didn't knew that so many things are possible on Facebook (social media).*
- *Everything we covered during the sessions was new to me and very interesting.*

- *My personal interest goes out to the PR and event-session.*

Nevertheless, the participants had suggestions on what could be change in the programme in the future. The top three suggestions were:

- *More practical exercises should be carried out (18.4%);*
- *Longer training period (16.3%); and*
- *More theory should be provided (14.3%).*

Only 4 participants did not want to change anything. All participants considered the trainers to be approachable. 95.9% reported that there was enough time for discussions during the training. 91.8% considered the provided material as sufficient. Table 6.3 presents the clarity of the future work post the workshops participation.

Table 6.3. Clarity of the future work post workshops

Question	Average score (Likert scale 1=worse to 5=best)
Are the objectives of the project campaign clear?	4.23
To which degree will the training help you to participate in the campaign?	4.02
Following the training are you confident in carrying out your part of the campaign?	4.10
Are the next working steps clear?	4.18

Despite the high ratings, the participants anticipated potential problems with most of them being related to overestimating the possible media reach and not reaching project goals. Proposed solutions spanned from setting reasonable goals through constant progress monitoring to increasing efforts.

6.3 QUE 3.1: Questionnaire at the first Project advisory groups meetings

As per the project timeline each partner established a Project advisory group (PAG) of road safety stakeholders. 48 PAG members, almost double the planned 25, submitted feedback forms after their first meeting. For breakdown by country see Figure 6.5.

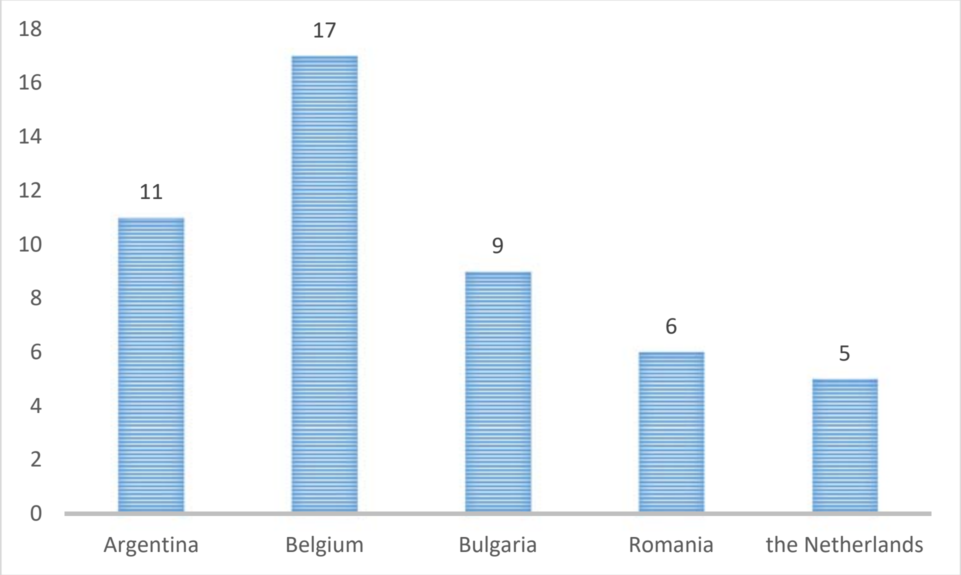

Figure 6.5. First meeting PAG participants per country.

The reported occupations were: Chief legal advisor regional roads managing; Head of Department of Administrative Services District government; Senior expert, Regional Governance of Education; Junior expert, Regional Health Inspection; Head Learning Centre, Union of Bulgarian Motorists; Instructor, Union of Automotive Instructors; Teacher; City councillor; Road safety expert; Driving instructor; Province councillor; Road Safety Organisation activist; Psychologist; Student; Press Officer - Buenos Aires City Legislature; Judicial officer in road accidents; Director of the Observatory and Statistic Unit of the Buenos Aires Province; Road safety Education Director on Buenos Aires City Government; Road safety GNO director and Road Safety program coordinator in La Plata City. Thus the representatives covered a variety of occupations and represented institutions. Their average age was 37 years. 30 of them were males, 18 were females. Table 6.4 reveals the average scores on the questions the PAG memebers were asked in regards to the clarity of the project.

Table 6.4. Feedback from PAG members in regards to the project design

Question	Average score (Likert scale 1=worse to 5=best)
Are the objectives of the project clear?	4.64
In your opinion, are the objectives achievable?	4.44
Are the working steps of the project clear?	4.46
Is the project timeframe appropriate?	4.38
Is your role in the project clear?	3.88

The only somewhat problematic area was the role of the PAG members in the project. Nevertheless, 40 PAG members stated that they were interested in following the projects during its full term and that they would be able to contribute to achieving its objectives. 29 of them committed to participating in a second Project advisory group meeting in 2018.

6.4 QUE 4: Questionnaire at the National Youth Summits on Road Safety

VAST was introduced to the local communities through National Youth Summits on Road Safety. They were organized by the project young road safety volunteers and were opened for active young people and youth workers who are interested in road safety. A common questionnaire was developed to collect the feedback of the participants in the Summits. It was translated in local languages to increase the likelihood for completion. Table 6.5 provides details on the provided feedback.

Table 6.5. Feedback from National Summits on Road Safety (scales from 1, worse, to 5, best)

	Argentina	Belgium	Bulgaria	Netherlands	Romania
Number of participants	53	51	67	50	52
Males	23	27	24	24	30
Females	30	24	43	26	22
Average age	33	25	28	22	27
Are the objectives of the VAST project clear?	4.66	4.39	4.64	3.98	4.62
In your opinion, are the objectives achievable?	4.45	4.00	4.87	3.56	4.35
Are the working steps of the project clear?	4.37	4.02	4.84	3.66	4.54
Do you think that the VAST campaign will produce a lasting change in the participants' views in regards to driving under the influence of drugs and/or alcohol?	4.53	3.45	4.87	3.56	4.33

With 273 collected feedback forms, the objective of involving 250 participants in National Youth Summits on Road Safety was achieved by the project partners. Furthermore, Table 6.5 reveals good gender balance as well as an average age in line with the intention to involve young people. It also shows that the Summits fulfilled their purpose. The project objectives were clear, also achievable, as were the project working steps with ratings above 4 for all countries with the exception of the Netherlands. There is also positive feeling about the added value the project will bring in regards to driving under the influence of drugs and/or alcohol, although the values are somewhat lower in Belgium and the Netherlands.

7. Evaluation 2: Campaign implementation

Table 7.1 gives an overview of the questionnaires which were used during the second evaluation phase.

Table 7.1. Overview of the questionnaires in evaluation phase 2

Action	Target group	Timing	Sample size	Questions regarding:
**EVALUATION 2: Campaign implementation – Summative approach**				
QUE 5: Questionnaire regarding the national implementation plans of the Joint Campaign	Country coordinators	Month 6 – December 2017	One from each partner – five in total	Evaluation of the implementation plan in each partner's context, the target groups and actions<br>Outline of the campaign<br>Planned actions including materials to be used<br>Target group
QUE 6: Questionnaire regarding the national implementation of the Joint Campaign	Country coordinators	Month 14 – August 2018	One from each partner – five in total	Evaluation of the campaign<br>Objective numbers regarding the campaign (number of actions, involved youth workers, contacted participants, distributed materials)<br>Satisfaction with the campaign<br>Possible problems
QUE 7: Questionnaire regarding the national implementation of the Joint Campaign	Youth workers and volunteer multipliers	Month 14 – August 2018	40 people (8 per partner country)	Evaluation of the effects of the campaign<br>Satisfaction with the campaign<br>Estimated satisfaction of the participants<br>Possible problems

QUE 8: Questionnaire regarding the national implementation of the Joint Campaign	Participants	Month 14 – August 2018	600 per partner country – 3000 in total	Evaluation of the effects of the campaign (TTM)<br>Satisfaction with the campaign<br>*Survey of the general public

QUE 5: Questionnaire regarding the national implementation plans of the Joint Campaign (Country coordinators)

After the planning for implementing the Joint International Road Safety Campaign, the country coordinators filled in a questionnaire with the main aim to gain an insight into the planned implementation and the wished for results of each partner and in particular the target groups, actions and dissemination activities. The results of the questionnaire were used to define indicators which were compared at a later stage with the actual achievements of the implementation. Furthermore, the overview of the planned implementations was used to develop specific questions for each campaign used in the subsequent evaluations.

QUE 6: Questionnaire regarding the national implementation of the Joint Campaign (Country coordinators)

At the end of the first implementation phase, the country coordinators filled in a questionnaire with the main aim to evaluate the effectiveness of the implementation of the Joint International Road Safety Campaign, the accomplishments and the dissemination of materials. The questions consisted of objective and subjective variables which can be compared with the indicators and objectives of the implementation plans to determine whether any problems occurred and if appropriate amendments have to be made in the future.

QUE 7: Questionnaire regarding the national implementation of the Joint Campaign (Youth workers and volunteers)

At the end of the Joint International Road Safety Campaign, the youth workers and volunteers filled in a questionnaire with the main aim to evaluate the effectiveness of the first implementation of the campaigns from their perspective. The results show their general satisfaction with their contribution to the campaign and their opinion regarding how the participants received the campaign. Furthermore, it highlights any problems that occurred and if appropriate measures were undertaken to overcome them.

QUE 8: Questionnaire regarding the national implementation of the Joint Campaign (Participants)

During the Joint International Road Safety Campaign, the participants filled in a questionnaire with the main aim to evaluate the effectiveness of the campaigns. The results reveal whether the campaign managed to

influence their salient beliefs. The questionnaire was translated into the languages of the participating countries, distributed during the implementation either directly online or in print and subsequently uploaded.

7.1.QUE 5: Questionnaire regarding the national implementation plans of the Joint Campaign

The country coordinators received a questionnaire with the request to complete it online after the planning of the implementation of the Joint International Road Safety Campaign. In the following Table 7.2, a summary of the results based on the questionnaire is presented.

Table 7.2. Implementation plans of the Joint International Road Safety Campaign

	Argentina	Belgium	Bulgaria	Netherlands	Romania
**Main Aims**	Raise awareness of the risk of driving under the influence of alcohol.	Create a safe experience of driving under influence while participants experience the potential dangers and risks for themselves.	To reach young drivers and raise their awareness on the dangers of driving under the influence.	Focus on the dangers of driving under influence of alcohol or drugs not just by telling participants about them but by letting them experience for themselves in our simulator.	Address a complex road safety problem, driving under the influence of alcohol and drugs.
**Campaign design**	We decided to do 15 implementations in schools and universities and 15 implementations in fairs, events, congresses.	In this campaign we want to target young drivers who have their permit or are up for their practical driving test. For the implementation we chose	Design was done by the members of the project team, of the NCSD board and of the project advisory group (PAG).	The whole year round we execute road-safety actions in the Netherlands. To target young drivers we had extra attention for events with a lot of young	1. Group meeting with volunteers and youth workers, figured out the basic elements of the campaign. 2. Meeting with PAG members, introduce the exercise, the

		May, because we can reach a lot of young students who are still on campus before exams start.		people present.	plans, problems and solutions.<br>3. Rewrite the campaign by using the advices of PAG members.<br>4. Fix the main elements of the campaign<br>5. Introduce our campaign in NYS, ask further recommendation and advices from the participants of NYS.
**Why this topic?**	It is the main cause of death among youngsters in Argentina.	This is an important topic for everyone. And mostly young drivers will benefit a lot because they are most at risk. For youngsters between 15 and 24 traffic is still the leading cause of death.	It is current and interesting.	We are very familiar with the dangers of drink- and drug-driving, and the risks of young drivers coming in contact with alcohol and drugs.	According to the Romanian police officers, this topic is a very important problem of our region, which should be addressed.

**Duration**	02/04/2018 - 15/11/2018	07/05/2018 – 30/09/2018	01/02/2018 - 01/07/2018	01/04/2018 - 30/09/2018	15/05/2018 - 31/08/2018
**Planned outreach**	600	200	550	1000	600
**Planned Actions**	Road Safety Stand in fairs, congresses and events. Road Safety workshops at schools and universities.	At least 4 VAST sessions at different campuses to assure a broad participants spectrum.	Interventions in schools, public authorities and public places.	We will organize a minimum of 10 VAST-actions (youth) events.	School campaign in secondary schools.
**Number of actions**	30	4	30	10	15
**Actions venues**	Universities, schools, Youth Olympic Games in Buenos Aires, fairs, conferences.	At several university and college-campuses in Flanders.	In all of the high schools in Vidin municipality, in the Traffic Police building, in the municipality hall, in the city center etc.	At different (youth) events across the Netherlands.	Oradea, Marghita, Baile Tusnad
**Tools**	Social media, banners, leaflets, games.	We will appeal to the schools communication channels to get our message across. Furthermore we will approach the student	Broad stakeholders' involvement: regional government of education, Traffic Police, regional Health	We will be in close contact with the event-organizer. We will try to make all attendees aware of our project before the event.	3D Virtual reality simulator, goggles, road safety short films, posters, leaflets, games

		councils on each campus and ask to propagate information about our campaigns.	Inspection and the media.		
**Involved youth workers and volunteers**	20	4	10	10	8
**Promotio-nal materials**	Leaflets	1000 information leaflets	Leaflets, brochures, t-shirts, souvenirs.	1000 leaflets	Leaflets, Posters, Pens, T-shirts, blotters.
**Announcing the campaign**	Internet and Social Media	Leaflets; Internet and Social Media; Intranet in participating campuses	Leaflets; Posters; TV Spots; Radio Spots; Newspaper Articles; Internet and Social Media	Leaflets; Internet and Social Media	Leaflets; Posters; Radio Spots; Internet and Social Media

7.2.QUE 6: Questionnaire regarding the national implementation of the Joint Campaign

The country coordinators received a questionnaire with the request to complete it online after the implementation of the campaign. The first part of the questionnaire focused on the campaigns implementation specifically while the second one looked into the dissemination activities that accompanied it. In the following Tables 7.3 and 7.4, a summary of the results is presented.

Table 7.3. Campaign implementation Argentina, Belgium and Bulgaria

	Argentina	Belgium	Bulgaria
Number of youth workers involved	5	4	3

Number of volunteers involved	12	4	7
Number of people reached directly	2,200	600	601
Number of people reached indirectly	6,600	500	104,697
Number of implemented actions	30	10	30
Campaign span	16/04/ – 13/11/2018	07/05/ - 30/09/2018	20/02/ - 13/06/2018
Overall satisfaction (out of 7)	7	5	7
Perceived satisfaction of involved participants (out of 7)	7	6	6
Do you think the campaign will help make the young people safer traffic participants?	Yes	Yes	Yes
Do you think the campaign could have been carried out with similar success but without the Virtual reality equipment?	No	No	No
How would you rate the impact of the virtual reality simulation on the involved participants?	7	7	6

Table 7.4. Campaign implementation in the Netherlands and Romania

	**Netherlands**	**Romania**

Number of youth workers involved	4	4
Number of volunteers involved	4	4
Number of people reached directly	1,000	900
Number of people reached indirectly	5,000	86,000
Number of implemented actions	14	9
Campaign span	01/04/ – 30/09/2018	29/03/ - 28/07/2018
Overall satisfaction (out of 7)	5	7
Perceived satisfaction of involved participants (out of 7)	6	5
Do you think the campaign will help make the young people safer traffic participants?	Yes	Yes
Do you think the campaign could have been carried out with similar success but without the Virtual reality equipment?	No	No
How would you rate the impact of the virtual reality simulation on the involved participants?	7	6

Most of the partners did not report any problems during the implementation of the campaign. Only in Romania lack of internet access which made it difficult to collect online evaluation forms was reported. The implemented solution was to use printed forms.

Dissemination activities were an important facet of VAST which is why the media capacity workshops were implemented at the beginning of the project. All countries were expected to use a variety of dissemination materials which is why the second part of the post-campaign questionnaire to the country coordinators focused on them. The country coordinators were asked to reflect on their dissemination activities with the request to provide data online. In the following Table 7.5, a summary of the results based on questionnaires is presented.

Table 7.5. Dissemination activities during the campaign implementation

	Argentina	Belgium	Bulgaria	Netherlands	Romania
Did you use dissemination materials during the campaign?	Yes	Yes	Yes	Yes	Yes
If you used dissemination materials, how many people you reached with them?	500	500	1,800	500	250
Did you use social media as part of the campaign?	Yes	Yes	Yes	Yes	Yes
If you used social media how would you rate the added value of the budget for social media? (out of 7)	6	5	6	5	6
If you used social media which social networks did you use?	Instagram, Facebook, Twitter	Instagram	Facebook	Facebook	Facebook
If you used social media how many people did you reach?	3,000	150	9,333	250	210
Did you use traditional media as part of the campaign?	No	No	Yes	No	Yes
If you used traditional media how would you rate the added value of the budget for the external PR experts?			6		6
Number of appearances in Internet media:		1	5	1	5
Number of appearances in newspapers:					4
Number of appearances on radio:					1
Number of appearances on TV:			4		2
Number of people reached through Internet media, excluding social media:		1,000	53,499	500	3,000
Number of people reached through newspapers:					30,000

Number of people reached through radio:					3,000
Number of people reached through television:			42,099		50,000

All partner countries provided information on their dissemination activities. Dissemination materials were used in all countries with 3,550 people being reached through them. The country coordinators rated the added value of the budget for social media at 5.6/7.0 and reported 12,943 people reached with social media. The added value of the budget for the external PR experts was rated at 6.0/7.0 by the two partners that reported using it. The following results were reported for traditional media:

- 12 appearances in Internet media = 57,999 people reached
- 4 appearances in newspapers = 30,000 people reached
- 1 appearance on radio = 3,000 people reached
- 6 appearances on TV = 92,099 people reached

7.3.QUE 7: Questionnaire regarding the national implementation of the Joint Campaign

The youth workers and volunteers, involved in the implementation of the Joined International Road Safety Campaign, received a questionnaire with the request to complete it online after the implementation of the campaign. In the following sections summaries of the reports are presented.

7.3.1. Argentina

16 people, 5 youth workers and 11 volunteers, provided their feedback in regards to the implementation in Argentina. 8 of them were females, 8 – males. The average age of the youth workers is 23 years, the one of the volunteers – 20 years. The youth workers and volunteers reported engaging in 6 actions on average or 102 in total.

Overall, their reported satisfaction with the implementation is 6.82/7.00. They also perceived very high satisfaction on behalf of the involved participants, 6.50/7.00. 13 of them believed that the campaign will help make the young people safer traffic participants, 3 thought it may do that. 13 of them believed that the campaign could not have been carried out with similar success but without the Virtual reality equipment, 3 thought it may have and 1 that it could have. The impact of the virtual reality simulation on the involved participants was rated at 6.69/7.00.

One youth worker reported encountered problems as well as implemented solutions: It was difficult to get all the people to fill the questionaries. ***Solution:*** We made more implementations.

7.3.2. Belgium

8 people, 4 youth workers and 4 volunteers, provided their feedback in regards to the implementation in Belgium. 4 of them were females, 4 – males. The average age of the youth workers is 25 years, the one of the volunteers – 22 years. The youth workers and volunteers reported engaging in 2 actions on average or 13 in total.

Overall, their reported satisfaction with the implementation is 6.25/7.00. They also perceived very high satisfaction on behalf of the involved participants, 6.38/7.00. Half of them believed that the campaign will help make the young people safer traffic participants, the other half thought it may do that. None of them believed that the campaign could not have been carried out with similar success but without the Virtual reality equipment. The impact of the virtual reality simulation on the involved participants was rated at 6.63/7.00.

No problems with the implementation were reported.

7.3.3. Bulgaria

8 people, 3 youth workers and 5 volunteers, provided their feedback in regards to the implementation in Bulgaria. 3 of them were females, 5 – males. The average age of the youth workers is 38 years, the one of the volunteers – 19 years. The youth workers and volunteers reported engaging in 7 actions on average or 55 in total.

Overall, their reported satisfaction with the implementation is 6.89/7.00. They also perceived very high satisfaction on behalf of the involved participants, 6.63/7.00. All of them believed that the campaign will help make the young people safer traffic participants and that it could not have been carried out with similar success but without the Virtual reality equipment. The impact of the virtual reality simulation on the involved participants was rated at 6.50/7.00.

No problems with the implementation were reported.

7.3.4. Netherlands

8 people, 4 youth workers and 4 volunteers, provided their feedback in regards to the implementation in the Netherlands. 5 of them were females, 3 – males. The average age of the youth workers is 25 years, the one of the volunteers – 22 years. The youth workers and volunteers reported engaging in 2 actions on average or 16 in total.

Overall, their reported satisfaction with the implementation is 6.13/7.00. They also perceived a high satisfaction on behalf of the involved participants, 6.25/7.00. Half of them believed that the campaign will help make the young people safer traffic participants, the other half thought it may do that. None of them believed that the campaign could not have been carried out with similar success but without the Virtual reality equipment. The impact of the virtual reality simulation on the involved participants was rated at 6.25/7.00.

No problems with the implementation were reported.

7.3.5. Romania

5 people, 2 youth workers and 3 volunteers, provided their feedback in regards to the implementation in Romania. 2 of them were females, 3 – males. The average age of the youth workers was 37 years, the one

of the volunteers – 22 years. The youth workers and volunteers reported engaging in 5 actions on average or 26 in total.

Overall, their reported satisfaction with the implementation is 6.80/7.00. They also perceived a very high satisfaction on behalf of the involved participants, 6.60/7.00. All of them believed that the campaign will help make the young people safer traffic participants. One of them believed that the campaign could not have been carried out with similar success but without the Virtual reality equipment, two through it could and the other two throught it might. The impact of the virtual reality simulation on the involved participants was rated at 6.00/7.00.

One youth worker and one volunteer reported encountered problems. Both reported the problems with filling in questioners with participants. ***Solution:*** We used printed sheets than bought a tablet.

7.4.QUE 8: Questionnaire regarding the national implementation of the Joint Campaign

A special focus of the evaluation is the long term effect of the campaign on the direct young participants in regards to assessing any shifts in their attitudes towards the risky behaviour of drink/drug driving. Psychological models were used to measure the success of the International Road Safety Campaign in regards to motivating safer behaviour in the campaign participants. The Theory of Planned Behaviour (TPB) (Ajzen, 1985, 1987, 1991) was selected as theoretical underpinning.

7.4.1. Theory of the Planned Behaviour

In psychology, the Theory of planned behaviour (See Figure 7.1) is a theory that links one's beliefs and behaviour.

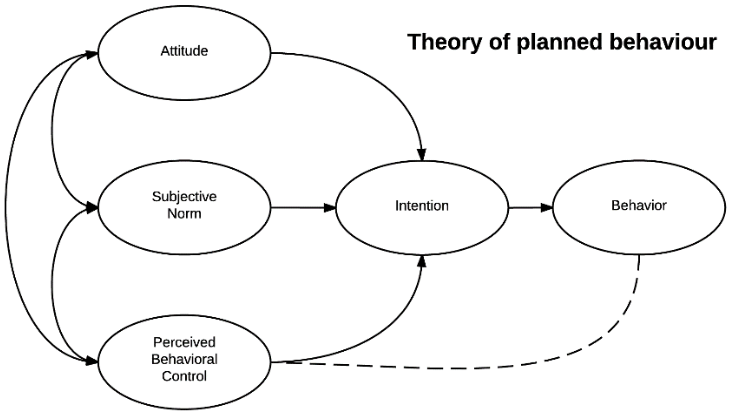

Figure 7.1. Theory of planned behaviour

TPB states that a person's attitude toward a particular behaviour, their subjective norms, and perceived behavioural control, together shape their behavioural intentions and behaviours. The concept was proposed by Icek Ajzen (1985, 1987, 1991) to improve on the predictive power of the Theory of reasoned action by

including perceived behavioural control. It has been applied to studies of the relations among beliefs, attitudes, behavioural intentions and behaviours in various fields such as advertising, public relations, advertising campaigns, healthcare, sport management and sustainability. This TPB was considered a good fit for VAST as the project targets drink- and drug-driving behaviour of young people and, on a higher level their intention, to perform it. As it is highly unlikely that many young people intend to drive under the influence of drugs or alcohol and even less likely to actually do so, VAST aimed at influencing the participants' attitude towards the behaviour.

7.4.2. Evaluation design

The intervention was designed as a randomised controlled trial (RCT). RTC is a trial in which subjects are randomly assigned to one of two conditions: one (intervention) in our case directly participating in the VAST campaign, and the other (control) which does not experience the virtual simulation. The two groups were to be followed up three months after the VAST campaign to see if there are any differences between them in taken measures. Measures were intended to be taken before and approximately three months post participation (See Table 7.6).

Table 7.6. Measures and time of measurement.

Construct	No. of items	Measured...		Source
		Before	After	
Demographic data	3	✓	✓	N.A.
Intention	2	✓	✓	
Attitude	2	✓	✓	
Subjective norm	2	✓	✓	Adapted from Elliott and Thomson (2010)
Perceived behavioural control	2	✓	✓	
DUI behaviour	1	✓	✓	

Driving under the influence of alcohol or drugs items, adapted from Elliott and Thomson (2010), were used to measure the TPB constructs using 9-point scales (scored 1–9). Intention to drive under the influence of alcohol or drugs (2 items) focused on 1) what the driver believes will happen and 2) what the driver believes is possible to happen in the other. Attitudes towards driving under the influence of alcohol or drugs (2 items) focused on drivers' 1) cognitive (instrumental) and 2) emotional (affective) attitude. Norms (2 items) explored 1) what would people who are important to driver think of them driving under the influence of alcohol or drugs (subjective norm); and 2) what those people will do themselves according to the driver (descriptive norm). Perceived behavioural control (2 items) explored 1) the drivers' own view of their

abilities (internal factors, self-efficacy) and 2) whether it would be possible to use those abilities in real life (external factors, perceived controllability). Driving under the influence of alcohol or drugs behaviour explored the drivers' recall of their actual behaviour in the recent past in regards to driving under the influence of alcohol or drugs. Example question: *To what extent do you intend to drive under the influence of alcohol or drugs over the next 3 months? (Possible answers: No extent at all (1) to A great extent (9))*

7.4.3. Recruitment of participants

Participants in the intervention group were recruited face-to-face by the project partner during the implemented VAST actions (See Table 7.7). They were incentivised to participate in the data collection before their participation by connecting the use of the virtual simulator with completing a survey. The follow-up survey did not offer incentives. A social media campaign was planned to be implemented at the beginning of the project in order to collect data from the general public through a survey, effectively establishing a baseline of a control group. The survey respondents were also contacted at the end of the campaign for second data collection. The Control group data was expected to be used to account for general shifts in people's attitudes due factors external to VAST.

Table 7.7. Number of collected participants' questionnaires per country, time and condition.

	Before the VAST campaign		Three months after the VAST campaign	
	Intervention	Control	Intervention	Control
Argentina	576	58	-	-
Belgium	141	42	-	-
Bulgaria	327	336	25	64
Netherlands	-	-	-	-
Romania	44	20	-	-

Unfortunately, three months after the VAST campaigns some data from the participants was successfully collected only in Bulgaria. Despite the efforts in the other countries, no success was achieved in that respect. Even the data from Bulgaria had problematic anonymous identifiers which hampered the intended analysis on top of the low numbers.

By all means, a quality set of post-intervention data from both the participants in the interventions and the Control groups was needed to complete the evaluation of the impact the project had on participants as originally intended. The lack of such data made performing the analysis impossible.

8. Evaluation 3: Dissemination activities

Table 8.1 gives an overview of the questionnaires which were used during the third evaluation phase.

Table 8.1. Overview of the questionnaires in evaluation phase 3

Action	Target group	Timing	Sample size	Questions regarding:

EVALUATION 3 Dissemination activities – Formative approach				
QUE 3.2: Questionnaire at the second Project advisory groups meetings	Stakeholders / PAG participants	Month 15 – September 2018	25 in total	Up-to-date project activities<br>Added value to the project<br>Future activities
QUE 9.1: Questionnaire at the first youth workers' mobility (13-26.09.2018)	Youth workers	Month 16 – September 2018	8 in total	Up-to-date project activities<br>Added value to the project<br>Logistics at the youth workers' mobility
QUE 10.1: Questionnaire at the European Road Safety Youth Summit	Stakeholders and participants in the European Road Safety Youth Summit	Month 16 – September 2018	35+ (Unlimited)	Evaluation regarding the project activities<br>Transferability of results<br>Satisfaction with the campaign<br>Possible problems and campaign improvement suggestions
QUE 9.2: Questionnaire at the second youth workers' mobility (12-25.11.2018)	Youth workers	Month 17 – November 2018	8 in total	Up-to-date project activities<br>Added value to the project<br>Logistics at the youth workers' mobility
QUE 10.2: Questionnaire at the South American Road Safety Youth Summit	Stakeholders and participants in the South American Road Safety Youth Summit	Month 17 – November 2018	25+ (Unlimited)	Evaluation regarding the project activities<br>Transferability of results<br>Satisfaction with the campaign<br>Possible problems and campaign improvement suggestions

8.1. QUE 3.2: Questionnaire at the second Project advisory groups meetings

The VAST project envisaged a second set of PAG meeting to be carried out upon completion of the Joint International Road Safety Campaign to share its achievements and to collect constructive feedback. The subsequent European and South American Youth Summits on Road Safety would had been introduced. After gathering for a second time PAG road safety stakeholders were asked to complete an online questionnaire.

A total of 38 PAG members submitted feedback forms after their second meeting. This kept the number of active road safety stakeholders within VAST above the planned 25. For breakdown by country see Figure 8.1.

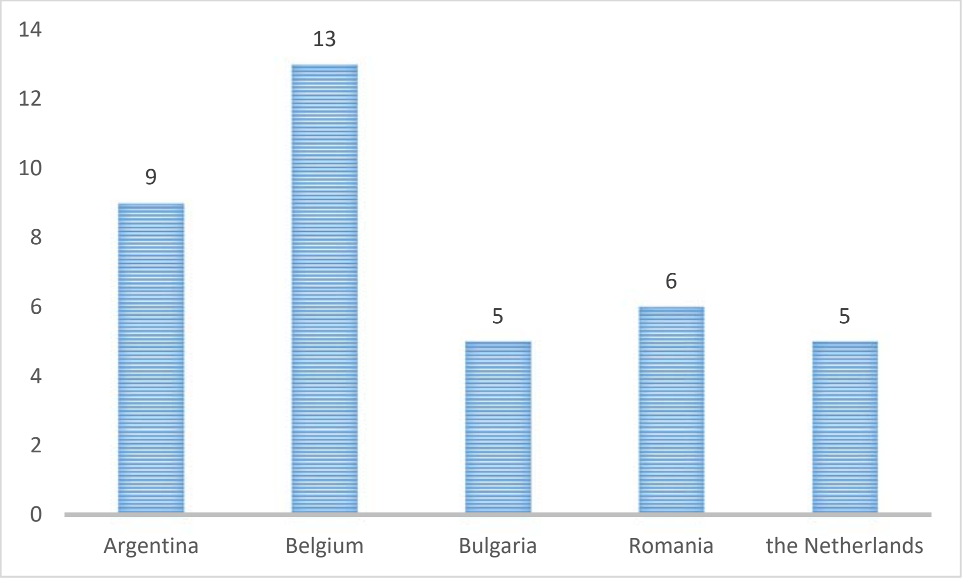

Figure 8.1. Second meeting PAG participants per country.

The reported occupations were: General Coordinator Road Safety Education - Buenos Aires City Government, Executive Director - Conduciendo a Conciencia Association, Judicial officer in road accidents, Student, NGO Assistant, Road Safety program coordinator in La Plata City, Road Safety Actor, City Council, Province council, Teacher, Insurance agent, Policeman, Road Safety Expert, Chief inspector automobile administration, Senior expert Regional department of education, Director of traffic police department – Vidin, Chief juris consult, Instructor, Doctor, Psychologist, Engineer, Public transportation and Road Safety Organisation. Again, the representatives covered a variety of occupations and represented institutions. Their average age was 37 years. 23 of them were males, 15 were females. Table 8.2 reveals the average scores on the questions the PAG memebers were asked in regards to the clarity of the project.

Table 8.2. Feedback from PAG members in regards to the project design

Question	Average score (Likert scale 1=worse to 5=best)

Are the objectives of the project clear?	4.61
In your opinion, are the objectives achievable?	4.21
Are the working steps of the project clear?	4.40
Is the project timeframe appropriate?	4.29
Is your role in the project clear?	4.05

The provided answers in regards to the project are similar to the ones given during the first PAG meeting and show consistent understanding of VAST. At this stage even a bigger percentage (87% in comparison with 83 during the first meeting) stated that they were interested in following the projects during its full term. It has to be noted though that the number of total people involved is less. 33 PAG members said they would be able to further contribute to achieving the VAST objectives. Probably the most important outcome of the PAGS is that 21 members committed to continue working as part of the Project advisory group beyond the project completion in December 2018.

8.2. QUE 9: Questionnaire at youth workers' mobility

Two two-week youth workers' mobilities took place in the VAST framework. To enlarge the added value for the project partners, each was represented by two youth workers and their participation took place in parallel with the European Youth Summit, organised by RYD BE, and the South American Youth Summit, organised by MiNU. The first youth workers' mobility took place in Brussels, Belgium, between 13 and26.09.2018. The second youth workers' mobility took place in Buenos Aires, Argentina, between 12 and 25.11.2018. Only the visiting youth workers' opinions were surveyed because the project does not subsidise host youth workers. They were asked questions in regards to the project in general and the youth workers' mobilities as a tool. 8 out of 8 participants completed the survey for the first mobility, 7 out of 8 for the second. Table 8.3 shows the collected results:

Table 8.3. Feedback from the youth workers' mobilities participants

Question	Youth workers' mobility in Brussels, Belgium	Youth workers' mobility in Buenos Aires, Argentina
Gender	Male: 6<br>Female: 2	Male: 4<br>Female: 3
Average age	33	34

Project		
Are the objectives of the project clear?	5.00/5.00	5.00/5.00
In your opinion, will the objectives be achieved by the end of the project?	4.88/5.00	5.00/5.00
Are the working steps of the project clear?	5.00/5.00	5.00/5.00
Was the project timeframe appropriate?	4.88/5.00	5.00/5.00
Do you think that such projects will produce a lasting change in the participating young people attitudes and/or behaviour on the road in regards to drink/drug-driving?	4.50/5.00	4.71/5.00
Do you believe that the VAST campaigns were successfully implemented?	Yes: 8	Yes: 7
Do you think that the campaign can be implemented with the necessary quality beyond the completion of VAST given the available resources?	Yes: 8	Yes: 7
Youth workers' mobilities		
Do you think "youth workers mobility" is a better replacement of a "partners' meeting only" in VAST?	Yes: 8	Yes: 6<br>No: 1
Do not think the youth workers' mobility added value to the hosting partner activities?	Yes: 5<br>Somewhat: 1<br>No: 2	Yes: 5<br>Somewhat: 0<br>No: 2
Do not think the youth workers' mobility added value to the VAST conference?	Yes: 5<br>Somewhat: 1	Yes: 5<br>Somewhat: 0

	No: 2	No: 2
How would you rate the youth workers' mobility programme?	4.88/5.00	4.86/5.00
How would you rate the youth workers' mobility accommodation?	4.63/5.00	4.57/5.00
How would you rate the youth workers' mobility food?	4.75/5.00	4.71/5.00
How would you rate the youth workers' mobility informal social aspect?	5.00/5.00	4.71/5.00
How would you rate the youth workers' mobility overall?	4.88/5.00	4.71/5.00

It is encouraging to see that the project design remained relevant throughout the project preparatory activities. The project partners' representatives were quite clear and very positive about the project process and the intended results.

The quality of the youth workers' mobilities was reported to be very high in any aspect with average scores very close or equal to the maximum possible 5. All respondents confirmed their appropriateness to be used as a framework for partners' meeting. Nevertheless, two participants shared doubt that the mobilities add value beyond the meetings, i.e. to the hosting partner or to the VAST conferences. This is an issue that would require in future endeavours increased effort on behalf of the project consortium to harvest the potential of delivering added value. However, the overall notion was positive in those aspects as well. A future evaluator should also keep in mind to look for underlying reasons for other-than-positive feedback to be able to provide better recommendations. Despite being given the opportunity to elaborate further, the two participants did not share details.

8.1. QUE 10: Questionnaire at the International conferences

The VAST Road Safety Summits took place in:

1. Brussels, Belgium, on the 22nd of September 2018,
2. Buenos Aires, Argentina, on the 16th of November 2018.

8.1.1. Brussels, Belgium

The European Road Safety Summit was implemented to disseminate the project experience from the PAGs meetings, the National Youth Summits on Road Safety and the Joint International Road Safety Campaign in Europe. To enlarge the added value for the project partners, each was represented by two youth workers

and their participation took the form of two-week youth workers mobility in support of the European Summit implementation for RYD BE (See Section 8.2). Stakeholders, young people and youth workers with interest in road safety youth work from European countries were invited to participate. The summit featured road safety best practices from South America.

50+ delegates attended the Summit. All VAST partners were represented. 37 of the delegates (23 male and 14 female) provided feedback. The average reported age was 32 years.

The participants were asked four questions related to the VAST project on a 1 to 5 Likert scale. All ratings were highly positive and above 4 with the exception of the potential for a long-term impact of the project. The last received a 3.75/5.00 rating which nevertheless is still very positive reflection on the project's added value. The average scores are presented in Table 8.4.

Table 8.4. European Road Safety Summit feedback

Question	Result
Are the objectives of the VAST project clear?	4.30
In your opinion, were the objectives achieved?	4.27
Are the working steps of the project clear?	4.30
Do you think that such projects will produce a lasting change in the participating young people attitudes and/or behaviour on the road in regards to drink/drug-driving?	3.76

Finally, the participants were asked to rate VAST overall in respect of its design, implementation, impact, sustainability. Again, the project received a positive rating of 3.72/5.00 which underlines its inherent qualities, something the project partners may wish to keep in future follow-ups. The participants were also asked to reflect specifically on the Joint International Road Safety Campaign implementation whether it was or was not successfully implemented. All participants pointed out its successful implementation.

8.1.2. Buenos Aires, Argentina

The second VAST Road Safety Summit was planned to take place in South America. Similar to the European one the intention was to disseminate the project experience from the PAGs meetings, the National Youth Summits on Road Safety and the Joint International Road Safety Campaign in South America. To enlarge the added value for the project partners, their representatives participated in the South American Summit through a two-week youth workers mobility, hosted by MiNU. Stakeholders, young people and youth workers with interest in road safety youth work from South American countries were invited to participate. The summit also featured road safety best practices from the European Union.

100+ delegates attended the Summit. All VAST partners were represented. 53 of the delegates (22 male and 31 female) provided feedback. The average reported age was 37 years.

The participants were asked the same four questions related to the VAST project on a 1 to 5 Likert scale as in the case of the European Summit. Again, all ratings were highly positive and above 4, a very positive reflection on the project added value. The average scores are presented in Table 8.5.

Table 8.5. South American Road Safety Summit feedback

Question	Result
Are the objectives of the VAST project clear?	4.55
In your opinion, were the objectives achieved?	4.38
Are the working steps of the project clear?	4.45
Do you think that such projects will produce a lasting change in the participating young people attitudes and/or behaviour on the road in regards to drink/drug-driving?	4.49

The participants were asked to rate VAST overall in respect of its design, implementation, impact, sustainability. The project received a very positive rating of 4.18/5.00 underlining its inherent qualities in the case of South America, too. The participants were also asked to reflect specifically on the Joint International Road Safety Campaign implementation whether it was or was not successfully implemented. 46 participants pointed out its successful implementation. 7 said it was successfully implemented to a certain extent.

9. Conclusions and suggestions

The targets set by the European Road Safety Policy Orientations 2011-2020 and the UN "Decade of Action on Road Safety (2011- 2020) of reducing road fatalities seem less and less likely to be reached. Technology gives hope for a higher outreach at a lower cost in prevention activities.

The VAST project partners possess massive experience in deploying Information technologies through peer-to-peer communication strategies. Through the project they put that experience to work and embraced the potential of next generation virtual reality technologies in an effort to address the overrepresentation of young people in road crashes. A tailored International campaign specifically designed to engage and appeal to contemporary and future generations of drivers was implemented to harvest the power of those technologies for social and public good.

The VAST country coordinators reported a successful implementation of the Joint International Road Safety Campaign in 93 separate actions by 20 youth workers and 31 young volunteers. The implementation was

rated at 6.20/7.00 by the country coordinators and at 6.60/7.00 by the youth workers and the young volunteers. The perceived satisfaction of the involved participants was 6.00/7.00 and 6.47/7.00 by the country coordinators and the youth workers and the young volunteers respectively. Unfortunately, due to low quality data, the effect on the participants themselves was not evaluated. This is where the main recommendation for continuing that excellent initiative comes. In future projects, if a second set of data is required from the participants, it may be more practical to look into the short-term effect of the campaign rather than the long-term one. Although, investigation of the long-term effect seems to be more valuable, VAST showed that in this specific case it was not achievable. On the other hand, while the participants were still on the campaign implementation site, they could had been asked to complete a second survey immediately after driving the VR simulator. It has to be acknowledged that such an approach was no part of the current evaluation design. Nevertheless, it is recommended to be used by evaluators of future similar initiatives.

Despite this shortcoming, the VAST reached 5,301 people directly and 202,797 people indirectly, numbers much higher than the initially planned ones. The overall rating of the impact of the virtual reality simulation on the involved participants was perceived at 6.49/7.00.

This successes could also be attributed partially to the high involvement of external stakeholders in VAST. 38 road safety stakeholders participated in the Project Advisory Groups from the project beginning through to its completion. Even more were involved in the project through the National Youth Summits on Road Safety (273 people), European Road Safety Summit (37 people) and the South American Road Safety Summit (53 people). VAST received an overall rating in respect of its design, implementation, impact, sustainability of 4.18/5.00 in Buenos Aires and 3.72/5.00 in Brussels with 92% of the International Summits' participants confirming its successful implementation.

Additionally the general public was well informed about this success with 3,550 people receiving dissemination materials in person, 12,943 people reached through social media and 183,098 people reached through conventional media.

It shall be expected that with the experience gained in VAST the project partners will be able to mainsteam the developed campaign into their regular activities. It may also be expected that the participating organizations are ready to build on their new capacity and follow-up VAST through larger joint activities. Thus, on top of the main recommendation descussed above in respect to collecting quality data for evaluating the impact of the project on the directly involved participants, some specific suggestions for futher work can be made:

1. Despite the implemented youth workers mobilities and their added value in VAST, no similar activities were planned or implemented on International level for the benefit of the young volunteers involved. A youth exchange may well suit the purpose in future projects.

2. A further option to enhanse the transfer of results would be creating a possibility for those being active in VAST (PAG members, project coordinators, youth workers and young volunteers) to all meet together, for example in an International road safety forum. This will provide an opportunity for sustainability.
3. A follow-up campaign implementation should be planned nationally if not Internationally not only to establish the VAST campaign but also to try to involve additional stakeholders, e.g. invite active road safety stakeholders from other countries to implement the campaign outside the current VAST geographical outreach. It would be a waste of involvement, knowledge and experience, if the acquired new capacity is not put to work in the future and subsequently expanded upon.

With the above listed suggestions in mind, this evaluation can be concluded with the following statements regarding the VAST project:

- ✓ VAST achieved its aims and objectives with one exception which we were unable to test for.
- ✓ The project methodology was found feasible and capable to contribute to road safety.
- ✓ The target group was appropriate and well defined.
- ✓ The campaigns effectively reached the target group.
- ✓ The project was implemented as planned.
