# Supplementary material for: Can’t simply roll it out: Evaluating a real-world virtual reality intervention to reduce driving under the influence
Source: PLoS One. 2021 Apr 29;16(4):e0250273. doi: 10.1371/journal.pone.0250273 (PMC8084189; doi:10.1371/journal.pone.0250273)
Supplement: Attachment — Submitted filename: Response to Reviewers.docx [file pone.0250273.s003.docx]

Reference: Can't simply roll it out: Evaluating a real-world virtual reality intervention to reduce driving under the influence [PONE-D-20-38963]

Dear editor,

Thank you for completing your evaluation of our manuscript, providing constructive feedback, and inviting us to revise the article!

We can confirm that we have:

1. Ensured the manuscript meets PLOS ONE's style requirements, including those for file naming.
2. Included captions for the Supporting Information files at the end of the manuscript, and updated any in-text citations to match accordingly.
3. Obfuscated the faces of the participants through image editing as advised by Mr. Jaime Gochez from the PLOS Editorial Office as a way to avoid the need for new consent forms. When the photographs were taken, we followed the standard QUT procedure and obtained written consent covering future use of the photos. We keep all collected consent forms, which can be presented upon request. However, we cannot determine which of the forms are relevant for the specific photos. Thus, we are unable to obtain PLOS Consent Forms for Publication in a PLOS Journal from the participants to have their images published under the Creative Commons Attribution (CC BY) license.

We have also revised the article according to the reviewers' comments. Track changes highlight all changes made in the manuscript. Here is how we addressed the comments of each reviewer:

Reviewer One

| Reviewer's Comments | Article Authors' Response | Page or other Reference |
| --- | --- | --- |
| Thank you for submitting to PLOS ONE. Regarding your submission, I have the following recommendations to strengthen the paper details and add scientific merit. Please revise the paper and clarify the following sections: | We thank the reviewer for the provided constructive guidance! |  |
| Apparatus and Context (Page 9):  Would the friends of the participants be distracting to the participants completing the task?  Was there any effort to control for this type of distraction?  Further the potential for student participants could be at risk of viewing the experiment details which could potentially confound the data? (priming effect).  Finally, were the participants given a training tutorial of how to use the HMD? | We thank the reviewer for the questions. We have provided the following clarifications:  "Although when present, such unintended casual discussions might have been distracting to the participants completing the task, no effort to control for this type of distraction was applied. It is not uncommon for young people to drive in the presence of others in their vehicles and to be commented on. Thus, the situation was considered representative of possible driving conditions, which aligned well with the experiment.  Nevertheless, it was considered that viewing a scenario before participation could potentially influence subsequent participants. Despite the fact that such priming effects, for example realising the level of reduced vision while under the influence of alcohol and adjusting head movements to compensate, may also appear when young people are on the road, an effort was made to mitigate potentially confounding the data by advising the viewers to try a scenario they have not seen when their turn comes. Such advice would be reiterated while the respective participant was introduced in detail to how to operate the simulator with the VR software." | Line 172 (Line 173 when All Markup is on) |
| VR intervention procedure (Page 9): Was an experimenter or PI present if the participant had questions? | We thank the reviewer for the question. We have provided the following clarification:  "A representative of the research team was present at all time to facilitate the intervention and answer any questions the participants might have had about the study or the simulator with VR software." | Line 186 (Line 187 when All Markup is on) |
| VR intervention procedure (Page 9): Please describe the VR software and what were its capabilities?  How were the scenarios presented using what platform? (e.g., Unity, Unreal).  Was a standard desk computer used? Or a computer with processing power capable of running the scenarios?  Were the participants debriefed in some manner following the final phase of the quasi - experiment? | We thank the reviewer for the questions.  The description of the VR software and its capabilities were part of the "VR intervention procedure", coming towards the end of the section. We have provided the following additional information:  "Once started, the software would position the participant as sitting into the driver's seat of a four-seat light vehicle with a clear view of natural night scenery. When turning their head around, the participant will be able to see a 360-degree view both in the car and of the outside scenery. In the vehicle, there is an empty front passenger seat and an empty back seat. The virtual car driving wheel will turn simultaneously together with the Logitech G29 driving wheel. A dashboard provides real-time feedback from the virtual car to the driver. Other feedback is received through the car mirrors, a big one in the centre of the windscreen and two at the sides. Generally, the interior looks like a real car. The only unrepresented thing is the participant, i.e. a participant cannot see their own body in the virtual environment. Upon the start of the software, the participant can hear the sound of turning the engine on. By default, the gear is put in automatic mode, ready to drive."  We have provided the following clarification at the first mention of the software in the body of the manuscript:  "A Unity-based".  We have provided the following clarification:  "The computer, a consumer-grade gaming desktop, had enhanced processing-power capabilities to be able to run the VR scenarios in full quality."  We have provided the following clarification:  "Although there was no planned debriefing session after a participant completed their VR simulation, the research team members were answering all questions the respective participant might have had." | Line 202 (Line 203 when All Markup is on)  Line 164 (Line 165 when All Markup is on)  Line 165 (Line 166 when All Markup is on)  Line 244 (Line 245 when All Markup is on) |
| Sample Size (Page 11): List females age range, Mean, Standard Deviation, and driving experience. Female biographical information may be listed and not made for the reader to infer. | We thank the reviewer for the provided suggestion! The listed descriptives refer to the whole sample, not to the males only. We believe that listing the gender-specific values for males and females in addition to the sample values may overwhelm and confuse the reader. This is why we prefer to leave only those whole-sample-relevant values in the narrative. Nevertheless, we changed "237 male" to "72% male", and subsequently "98 male" to "66% male" in an effort to reduce any confusion. | Line 250 (Line 251 when All Markup is on) |
| Means, Standard Deviation and Bivariate Correlations (Page 15): Consider listing Spearman’s correlations in the body of text (first sentence). It is unclear. | We thank the reviewer for the provided suggestion! We have listed the Spearman’s correlations in the body of the text. | Line 298 (Line 317 when All Markup is on) |

Reviewer Two

| Reviewer's Comments | Article Authors' Response | Page or other Reference |
| --- | --- | --- |
| This paper attempts to measure the effectiveness of a public VR DUI simulator as an intervention to reduce intent to DUI through a quasi-experiment involving adults 18-25 aged 18-25 with no history of DUI. The authors show no significant effect of the intervention on DUI intent and self-reported DUI behavior.  STRENGTHS  This paper is very thorough in defining and showing data, data analysis methods, and procedures. It is very clear what the authors did to achieve these results. The authors also succeeded in recruiting a large sample size, relative to many VR studies concerning road safety. | We thank the reviewer for the nice words! |  |
| KEY WEAKNESSES  Participants are individuals that have no recorded history of DUI and generally self-report no DUI behavior. It appears flawed to use individuals that would not DUI in the first place to assess the quality of any intervention for reducing DUI behavior. For example, in Elliott and Thomson's work that the authors cite as a basis for the survey questions, they specifically target drivers with demonstrated speeding history when assessing the validity of their behavior model. It is hard to say whether they could have achieved the same results if all of their participants simply reported little or no intention to speed.  It is therefore difficult to use the given quasi-experiment to question the utility of VR interventions for DUI, as it appears that any intervention would be ineffective on a population that was not intending to exhibit the behavior in question. The authors indeed acknowledge this in their "Findings" section. Yet, in their abstract and conclusion, the authors do not do the same, creating the impression that there was some flaw in the VR intervention itself or their survey methodology that lead to the results.  CONCLUSION  Due to the above mismatch between what appears to be the primary cause for the null results and the claims the authors make about VR interventions for DUI, I recommend revising the paper to be more clear about this fundamental fact of the experiment. As a reader, I felt confused for most of the paper after reading in the abstract that the participants had no history of DUI, but then feeling like this fact went unaddressed for the majority of the paper thereafter. This paper would appear to fully meet the intention of this journal if the conclusions matched what appears to be a clear picture painted by the data. | We agree with the reviewer's comprehensive comment.  To improve the clarity of the Abstract, we have replaced:  "Such results question the utility of funding the roll-out of arguably attractive technologies without a thorough understanding of their effectiveness. In particular, we provide suggestions on how VR software might be further developed and, subsequently, leveraged in future research to improve the potential of future interventions for behavioural change, e.g. by collecting, analysing and presenting objective driving performance data."  with  "Such results might be due to the fact that the recruited participants generally self-reported little DUI behaviour, i.e. positively changing behaviour that is already positive is inherently challenging. Nevertheless, the results question the utility of funding the roll-out of arguably attractive technologies without a thorough understanding of their effectiveness in particular settings. To improve the potential for future positive outcomes of such interventions, we provide suggestions on how VR software might be further developed and, subsequently, leveraged in future research to improve the likelihood for behavioural change, e.g. by collecting, analysing and presenting objective driving performance data. Alternatively, future intervention might focus on participants with known DUI history and examine the effects of the VR intervention for this particular higher-risk group."  In the hypothesis, we have clarified  "without a known record of DUI"  by expanding the explanation to  "without preliminary information about any known records of DUI"  To improve the understanding that the participants were randomly recruited, we have added to the description "targeted recruitment, pre-screening".  In the Conclusion, we have expanded:  "Overall, this study found that the implemented VR intervention did not trigger a statistically significant shift in the participants' self-reported Past DUI behaviour and DUI intention. At the same time, a number of ways to increase the potential of VR as an intervention tool were identified, which should help both researchers in future studies and practitioners in future applied projects. Such suggestions should be considered before VR interventions are rolled-out on a large scale."  into  "Overall, this study found that the implemented VR intervention did not trigger a statistically significant shift in the participants' self-reported Past DUI behaviour and DUI intention. Nevertheless, we do not question the utility of VR interventions for DUI in general, but rather the utility of this specific VR intervention for DUI as currently implemented by being installed in a public space. In such open-public opportunistic interventions, it is unlikely that there will be preliminary information about whether individuals have a recorded history of DUI. Similar to the current study, such self-reported information may become available after the data is analysed. Obtaining such information in advance may be the key to unlocking the potential of VR interventions for DUI for triggering positive behavioural change. If such information is available before an experiment takes place, researchers might find it beneficial to focus the intervention on young drivers who report DUI.  The null results within the current study might be due to a large extent to the fact that the recruited participants generally self-reported little DUI behaviour. This objective outcome may question the utility of funding the roll-out of arguably attractive technologies without a thorough understanding of their effectiveness in particular settings. To overcome such shortcomings, a number of ways to increase the potential of VR as an intervention tool were identified, such as real-time data collection and feedback delivery. If implemented, those suggestions should help both researchers in future studies and practitioners in future applied projects. Thus, they should be considered before VR interventions are rolled-out on a large scale." | Line 27  Line 118 (Line 119 when All Markup is on)  Line 121 (Line 122 when All Markup is on)  Line 450 (Line 469 when All Markup is on) |
| OTHER TARGETS FOR REVISION  The authors performed a great bit more data analysis than the hypothesis and other content before the "Analysis" section would lead readers to expect. It would greatly clarify the intent and content of the paper if the authors informed the readers that they also intended to explore other correlations that could be found in the data. | We thank the reviewer for the provided suggestion! To clarify the intent and the content of the paper, we have integrated the content of the "Data transformation" section into the "Analysis" section. Additionally, we have added the following explanations:  "Initially, its descriptive statistics (means and standard deviations) were examined. To extract preliminary information about the relationships between the four main variables (DUI intention at T1, DUI intention at T2, Past DUI behaviour at T1, and Past DUI behaviour at T2), Spearman's rho correlation coefficients were calculated along with their significance levels." | Line 265 (Line 266 when All Markup is on) |

Thank you again for your interest in our paper!

On behalf of the co-authors of this paper,

Yours sincerely,

Dr Daniel Vankov

Centre for Accident Research & Road Safety - QLD

Queensland University of Technology

K Block, 130 Victoria Park Road, Kelvin Grove, QLD 4059

t: +61 7 3138 7727 e: d.vankov@qut.edu.au
